# The *VAChT^Y49N^* mutation provides insecticide-resistance but perturbs evoked cholinergic neurotransmission in *Drosophila*

**DOI:** 10.1371/journal.pone.0203852

**Published:** 2018-09-11

**Authors:** Samuel W. Vernon, Jim Goodchild, Richard A. Baines

**Affiliations:** 1 Division of Neuroscience and Experimental Psychology, School of Biological Sciences, Faculty of Biology, Medicine and Health, University of Manchester, Manchester Academic Health Science Centre, Manchester, United Kingdom; 2 Syngenta Crop Protection Research, Bracknell, Berkshire, United Kingdom; EPFL, SWITZERLAND

## Abstract

Global agriculture and the control of insect disease vectors have developed with a heavy reliance on insecticides. The increasing incidence of resistance, for virtually all insecticides, threatens both food supply and effective control of insect borne disease. CASPP ((5-chloro-1’-[(E)-3-(4-chlorophenyl)allyl]spiro[indoline-3,4’-piperidine]-1-yl}-(2-chloro-4-pyridyl)methanone)) compounds are a potential new class of neuroactive insecticide specifically targeting the Vesicular Acetylcholine Transporter (*VAChT*). Resistance to CASPP, under laboratory conditions, has been reported following either up-regulation of wildtype *VAChT* expression or the presence of a specific point mutation (*VAChT*^*Y49N*^). However, the underlying mechanism of CASPP-resistance, together with the consequence to insect viability of achieving resistance, is unknown. In this study, we use electrophysiological characterisation of cholinergic release at *Drosophila* larval interneuron→motoneuron synapses to investigate the physiological implications of these two identified modes of CASPP resistance. We show that both *VAChT* up-regulation or the expression of *VAChT*^*Y49N*^ increases miniature (mini) release frequency. Mini frequency appears deterministic of CASPP activity. However, maintenance of SV release is not indicative of resistance in all cases. This is evidenced through expression of syntaxin or complexin mutants (*sytx*^*3-61*^*/cpx*^*SH1*^) that show similarly high mini release frequency but are not resistant to CASPP. The *VAChT*^*Y49N*^ mutation additionally disrupts action potential-evoked cholinergic release and fictive locomotor patterning through depletion of releasable synaptic vesicles. This observation suggests a functional trade-off for this point mutation, which is not seen when wildtype *VAChT* is up-regulated.

## Introduction

Vesicular transporters load neurotransmitter into synaptic vesicles (SV) for storage before release. Transporter localisation dictates loading substrate and differs between vesicle classification [1]. Small clear SVs mostly store fast-acting neurotransmitters. Transporters known to localise to small clear SVs include the vesicular glutamate transporters *VGLUTs 1–3* [2–4], the glycine transporter *VGLYT1* [5, 6], the vesicular GABA and Glycine co-transporter *VGAT/VIAAT* [7–9], *VAChT* [10] and vesicular monoamine transporters (*VMATs*) [11]. The insect CNS and to a lesser, but still significant, extent the mammalian CNS relies on ACh and *VAChT* for excitatory synaptic signalling. *Drosophila VAChT* null mutants die during embryogenesis [12] whilst mammalian *VAChT* null mutants are lethal soon after birth [13]. *VAChT* knockdown also results in memory deficits in both mice and insects [14, 15]. By contrast, *VAChT* up-regulation causes accelerated neuromuscular aging [16] and disrupted central cholinergic morphological development [17]. Altered physiological and morphological phenotypes are also reported in *Drosophila* at the glutamatergic neuromuscular junction (NMJ) following *VGLUT* up-regulation [18]. It is, perhaps, unsurprising that transporter abnormalities are associated with many neurodegenerative diseases including Alzheimer’s [19], Huntington’s [20] and Parkinson’s disease [21].

Cholinergic central interneurons form a central pattern generator that drives *Drosophila* motoneurons and are pivotal in governing the control of larval peristalsis [22]. Normal cholinergic function is essential for survival and, as a result, the cholinergic system is a major target for insecticide development [23]. CASPP compounds specifically bind to, and inhibit the function of, *VAChT* [24–29]. CASPP mortality is associated with an inability of central neurons to release ACh [30]. However, transgenic up-regulation of *VAChT* significantly reduces CASPP lethality in adult *Drosophila*, to an extent greater than observed in *C*. *elegans* [25] and, moreover, greatly increases spontaneous mini release of ACh from larval *Drosophila* central premotor interneurons [30]. This suggests a causal relationship between up-regulation of target activity and resistance to CASPP. Furthermore, transgenic expression of the *VAChT*^*Y49N*^ mutation, in an otherwise wildtype background, provides complete insensitivity to CASPP [25]. However, the physiological implications to CNS function of the presence of this resistance allele, and its response to insecticide treatment, have not been described.

Insecticide resistant phenotypes have been formally identified since the discovery of DDT resistance in *Musca domestica* [31] and have been characterised into four sub categories; (i) behavioural changes, (ii) altered penetration, (iii) target site modification and (iv) metabolic resistance [32]. Through these mechanisms, insects have attained resistant alleles for the majority of pesticide classes including but not limited to organophosphates, carbamates, pyrethroids, neonicotinoids and non-neuronal insecticides such as insect growth regulators (IGRs) [33–36]. However, little is known about the physiological implications brought about by resistant genotypes. Here we utilise *Drosophila* as a model to study CASPP resistance generated through transgenic up-regulation of wildtype *VAChT* transcript achieved via the GAL4/UAS system and through the endogenous expression of a CRISPR-induced knock-in of *VAChT*^*Y49N*^. Electrophysiological characterisation of cholinergic minis in identified *Drosophila* motoneurons (specifically the anterior corner cell, aCC and Raw Prawn 2, RP2 motoneurons) shows that CASPP resistance is associated with the maintenance of mini release frequency. However, despite trends in enhanced synaptic transmission, we further demonstrate that similarly increased mini release, achieved through up-regulation of mutant membrane bound SNARE protein syntaxin *(sytx*^*3-69*^*)* [37] or the absence of complexin *(cpx*^*SH1*^*)* [38], does not lead to CASPP-resistance. This suggests that resistance to this class of insecticide cannot be fully explained by enhanced mini release at cholinergic synapses. Perturbation of evoked release (i.e. action-potential dependent) associated with *VAChT*^*Y49N*^ additionally disrupts the ability of the cholinergic locomotor system to maintain sustained activity attributed by an apparent change in SV release. These deficits translate into fitness costs observed through abnormalities in locomotion and adult longevity.

## Materials and methods

### Fly stocks

Flies were maintained under standard conditions at 25°C. GAL4 drivers used to recapitulate expression of the cholinergic locus were *cha*^*B19*^ [39] and *ChAT-BAC* (gifted by Steve Stowers: Montana State University). These lines were used to drive expression of UAS-*VAChT*, UAS-*sytx*^*3-69*^ (syntaxin mutant generously provide by Bing Zhang, University of Missouri) or UAS-*ChR2*^*ChETA*^ (Bloomington 36354) [40]. The complexin mutant (*cpx*^*SH1*^) was generously provided by Troy Littleton (Massachusetts Institute of Technology). The wild-type UAS-*VAChT* is described in [30]. CRISPR constructs were prepared as described below and injected into c*as9*-expressing embryos (*yw*; attP40{*nos*-*cas9}* /*CyO*;+) by BestGene Inc., (Chino Hills, CA, USA). Control lines were Canton-S and the CRISPR-injected line lacking construct insertion (*yw*; attP40{*nos*-*cas9}*;+).

### gRNA and insert design, template oligo and plasmid construction

The CRISPR Optimal Target Finder tool (http://tools.flycrispr.molbio.wisc.edu/targetFinder/) was used to specify target cut sequence specificity (GATTACCGCTATCAGGTACC). Two guide RNA constructs were made to generate cuts in 5’- and 3’-UTR of *VAChT*, respectively. The gRNA oligonucleotides (5’ to 3’) are: 5’-UTR: CTTCGAGAGGAAGTCCCAAAGAAAC and AAACGTTTCTTTGGGACTTCCTCTC; 3’-UTR: CTTCGTATTATTACTATAGACATAT and AAACATATGTCTATAGTAATAATAC, sense and antisense, respectively). 100 pmol of each 5’ phosphorylated sense and antisense gRNA oligonucleotides were mixed, denatured at 95°C and then reduced to 25°C at a rate of -0.1°C/sec and ligated to the guide RNA expression plasmid, pU6-BbsI-chiRNA (plasmid #45946, addgene). A *VAChT*^*Y49N*^, containing 5’ and 3’ PAM site mutations, that prevent cas9 cleavage, was cloned to pHD-DsRed vector (plasmid #51434, addgene) as a dsDNA donor template for CRISPR/Cas9-mediated homology-directed repair (HDR). Oligos used to generate PAM site mutations are depicted in Table 1. Briefly, for 5’ PAM site mutagenesis, PCR of primers a+b and c+d (containing TGG to TGC point mutations) were run against *Drosophila* genomic DNA (PCR1). Following purification, PCR products (a+b and c+d) were used as templates for a second PCR using the most 5’ and 3’ primers of PCR1 (primers a and d, Table 1). This process was repeated for 3’ PAM site mutagenesis utilising primers (e+f and g+h). Full UTR sequence with PAM mutations were purified, sequenced and mobilised to pHD-DsRed using restriction digests (5’ = AscI & BssSI), 3’ = SpeI & XhoI). *VAChT*^*Y49N*^ ORF was generously provided by Syngenta and cloned into pHD-DsRed vector using restriction enzymes (EcoR1 and Nde1). Sequence confirmation was confirmed by Sanger sequencing at the Manchester Sequencing Facility.

**Table 1:** Primers used for creation of *Drosophila VAChT* UTR with modified PAM sites (5’: a,b,c,d and 3’: e,f,g,h).

Sequence	Use
ATCGGGCGCGCCGAATTCATGCTTGGGTCGACTTAAGCTC	a
ACAAAGTTCTGATGCAGTTTCTTTGG	b
CCAAAGAAACTGCATCAGAACTTTGT	c
CTTAAATAGTCGGGTATAATCGGTACTA	d
GTACACTAGTTCGTGTTCTTTTGCACACCTCC	e
ACGTACCACTTGGCTATATGTCTATA	f
TATAGACATATAGCCAAGTGGTACGT	g
GCTACTCGAGAAGTCCGCCACAATGACAACC	h

### Identification of positive progeny

Positive progeny were identified by the expression of DsRed in larvae following the 3xP3 expression pattern. Single adult males were selected and individually balanced using *CyO* and *TM3*. Fully balanced lines were sequenced for the *VAChT*^*Y49N*^ sequence. DsRed was removed using a Cre-recombinase line (*y*^*1*^*w*^*67c23*^ *P {Crey} 1b*; D*/TM3, *Sb*^*1*^, flybase ID: FBst0000851). Lines were then homozygosed using (*w*^-^; *if/CyO*; *MKRS/TM3*) and cleaned generating a final stock of *w*^-^; +; *VAChT*^*Y49N*^.

### Mortality and 5-Cl-CASPP application

5-Cl-CASPP (also termed Syn351), made by Syngenta [25], was added to food vials containing standard cornmeal medium. 5-Cl-CASPP was solubilized in acetone (which was added alone as vehicle control). CASPP solution (50μl) was added to each vial and blunt forceps were used to break up the food surface to aid penetrance. A total of 60 first instar larvae were assayed per drug concentration, 20 larvae per vial. Larvae were reared at 25°C for 7 days (in a humid incubator). Mortality rate was measured as the proportion of larvae developing to pupae, against those that did not. For use in electrophysiology assays, 5-Cl-CASPP (10μg/ml) was added to the surface of grape-agar plates (50 mm diameter) in 1 ml of aqueous dried yeast extract (5%; Merck, Darmstadt, Germany). After being left to dry overnight at room temperature, second instar larvae were allowed to feed for 24 h before recording.

### Larval whole-cell patch-clamp recordings

Recordings were performed at room temperature (20–22°C). Third-instar larvae were dissected in external saline (in mM: 135 NaCl, 5 KCl, 4 MgCl2·6H2O, 2 CaCl2·2H2O, 5 N-Tris[hydroxymethyl]methyl-2-aminoethanesulfonic acid, and 36 sucrose, pH 7.15). The CNS was removed and secured to a Sylgard (Dow-Corning, Midland, Michigan, USA)-coated cover slip using tissue glue (GLUture; WPI, Hitchin, UK). The glia surrounding the CNS was partially removed using protease (1% type XIV; Sigma, Dorset, UK) contained in a wide-bore (15 μm) patch pipette. Whole cell recordings were carried out using borosilicate glass electrodes (GC100TF-10; Harvard Apparatus, Edenbridge, UK), fire-polished to resistances of between 8–12 MΩ. The aCC/RP2 motoneurons were identified by soma position within the ventral nerve cord [41]. When needed, cell identity was confirmed after recording by filling with 0.1% Alexa Fluor 488 hydrazyde sodium salt (Invitrogen, Carlsbad, California, USA), included in the internal patch saline (in mM: 140 potassium gluconate, 2 MgCl2·6H2O, 2 EGTA, 5 KCl, and 20 HEPES, pH 7.4). Tetrodotoxin (TTX, 2 μM, Alomone Labs, Hadassah Ein Kerem, Israel) was included in the external saline to block action potential-induced SV release. Recordings were made using a MultiClamp 700B amplifier. Cells were held at -60 mV and recordings were sampled at 100 kHz and lowpass filtered at 0.5 kHz, using pClamp 10.6 (Molecular Devices, Sunnyvale, CA). Only neurons with an input resistance of ≥ 500 MΩ were accepted for analysis.

Evoked vesicle exocytosis, mimicking action potential mediated spontaneous rhythmic currents (SRCs), were elicited through driving UAS-ChR2^Cheta^ (λ470 nm, 10ms, 1Hz/10Hz) using the cholinergic driver ChAT-BAC in the background of the wild type *VAChT* or mutant *VAChT*^*Y49N*^. Stimulations were carried out for up to 1 hour. As is true for other cholinergic drivers (e.g. *cha*^*B19*^-GAL4), ChAT-BAC shows weak expression in aCC (our unpublished observations). Recordings conducted in the presence of the nAChR antagonist, mecamylamine, reveal a small ~20pA inward depolarising current in unison with stimulating light pulses (i.e. due to this weak expression). Therefore, events ≤ 20pA, measured using the Clampfit threshold search function, were considered a failed event and thus the point at which the vesicle pool had been depleted.

### Larval extracellular recordings

Bursts of spiking driven by cholinergic synaptic input in aCC/RP2 motoneurons were investigated using loose patch extracellular recordings. Recordings were conducted using a 1.3MΩ recording electrode filled with extracellular solution allowing a loose seal on the soma when applying gentle suction. Recordings, sampled at 20 KHz, were made for 3 minutes with the second minute of each recording taken for analysis. The number of activity bursts within this second minute of recording, together with the number of spikes per burst (for the first 10 bursts) were quantified and averaged for each recording. Bursts were defined as a minimum of three events within 25 ms.

### Adult giant fibre activation

Flies were sedated on ice for 5 mins before being mounted in dental wax (Kemdent, UK). Appendages, wings and proboscis were secured into the wax with thorax being positioned at a 45° angle aiding penetration of recording electrodes. Two sharpened tungsten wires (0.2mm) were inserted into the CNS through each compound eye whereas the third was positioned into the abdomen. Stimulation was conducted with a SIU5A stimulus isolation unit (Grass Technologies, USA) controlled via a S88 stimulator (Grass Technologies, USA). Positioning of both stimulating and earth electrodes was confirmed with a pulse (50V, 0.02 ms) through observation of wing twitching (DLM stimulation) and tergotrochanteral muscle contraction (TTM stimulation). Intracellular recording electrodes (20–30 MΩ) (GC100FS-10; Harvard Apparatus, WPI, USA) were filled with 3M KCl for recording. Glass recording electrodes were inserted into DLM 45a and the TTM, identified by position of the bristles on the thorax. Recordings were performed using an Axoclamp-2A amplifier (Axon instruments, USA) controlled by pClamp 10.4 and a Digidata 1440A (Molecular Devices, USA). The responsiveness of the respective muscle (DLM and TTM) to a train of 10 stimuli at 100Hz, termed “Following Frequency” were recorded.

### Larval crawling

Single larvae were placed on a 55 x 34mm, 2% nutrient free agarose island surrounded by 5M NaCl to prevent escape. Recordings were conducted for 3 minutes using EthoVision XT (Version 11, Noldus, Wayeningen, Netherlands) in a Daniovision Behavioural Chamber (Noldus, Wayeningen, Netherlands), using a Basler GenIcam (Basler acA1300-60, Resolution: 1280x960, Frame Rate: 15Hz). Background was adjusted for using the inbuilt dynamic subtraction function, and a smoothing function of 10 was applied (each data point was a function of 10 data points averaged). Larvae were considered moving when breaching a 0.30mm distance threshold. Recordings were discharged if sample detection threshold was ≤15% of total recording. Mean velocity (mm/s) and total distance covered (mm) within each 3 minute recording were taken for analysis.

### Oviposition and longevity

Number of eggs laid per mated female was taken as one measure of overall fitness. 10–20 virgin females were allowed to mate for 48 hours under standard rearing conditions before being transferred to egg laying chambers mounted on a grape-agar plate (50 mm) supplemented with yeast paste. Following a 24 hour period, total number of embryos per plate was quantified. Following oviposition, single mated females from each genotype were added to single vials and allowed to lay for 5 days. The presence of viable larvae was considered proof the female had been mated. Total life span was further taken as a measure of general fitness. Newly eclosed male flies were added to standard rearing vials at a density of 10 flies per vial per replicate. Flies were transferred to fresh medium three times per week and deaths recorded, as per [42].

### Quantitative RT-PCR (qRT-PCR)

Ten CNSs were collected from third-instar wall-climbing larvae. RNA was extracted using the RNeasy micro kit (QIAGEN, Manchester, UK). Single strand cDNA was synthesized using the Revert Aid^™^ H minus First strand cDNA synthesis kit (Fermentas, Massachusetts, USA). qRT-PCR was performed using a LightCycler480 II (Roche, Basel, Switzerland) with SYBR Green I Master reaction mix (Roche, Basel, Switzerland). The thermal profile used was 10 min at 95°C followed by 45 cycles of 10 s at 95°C, followed by 10 s at 60°C, and finally 10 s at 72°C. Single-product amplification was confirmed by post-reaction dissociation analysis. PCR primers were designed with the aid of LightCycler Probe Design Software 2.0 (v1.0) (Roche, Basel, Switzerland). Results were analysed by the 2^-ΔCt^ method where ΔCt was determined by subtracting the average *rp49* Ct value from that of *VAChT*. Ct values used were the means of four to five independent repeats of 10 CNS per sample. Control gene was *rp49*. Primers (5’ to 3’) were as follows: *rp49*, CCAGTCGGATCGATCGATATGCTA and ACGTTGTGCACCAGGAA; *VAChT*, CTCATCCTCGTGATTGTA and ACGGGTATGATCTTTCC.

### Statistics

LD_50_ values were calculated by fitting a regression analysis to the relationship between % mortality and log[dose] using the following equation: $Response=\frac{100}{1+10^{\left(Log{LD}_{50}-X\right)}}$. Statistical significance between group means was assessed using either a Student’s t-test (where a single experimental group is compared to a single control group), a one-way ANOVA followed by Bonferroni’s post-hoc test (multiple experimental groups) or a Chi-Square test. In all tests, confidence intervals of *P≤ 0.05, **P ≤ 0.01, and ***P ≤ 0.001 and **** P<0.0001 were used for significance. Data shown is mean ± s.e.m.

## Results

### Increased *VAChT* or the presence of *VAChT*^*Y49N*^ infers resistance to 5Cl-CASPP

Transgenic up-regulation (using GAL4/UAS) of either *VAChT* or *VAChT*^*Y49N*^, in a wildtype background, is sufficient to provide resistance to 5-Cl-CASPP [25]. To validate the previously published observation of 5-Cl-CASPP resistance when *VAChT* is up-regulated, and to ascertain resistance associated with endogenously expressed *VAChT*^*Y49N*^, mortality assays were conducted in larvae where *VAChT* was up-regulated in all cholinergic neurons (*cha*^*B19*^>*VAChT*) or expression of *VAChT*^*Y49N*^ achieved through CRISPR knock-in (*VAChT*^*Y49N*/*Y49N*^ and *VAChT*^*Y49N*/+^).

**Fig 1:**
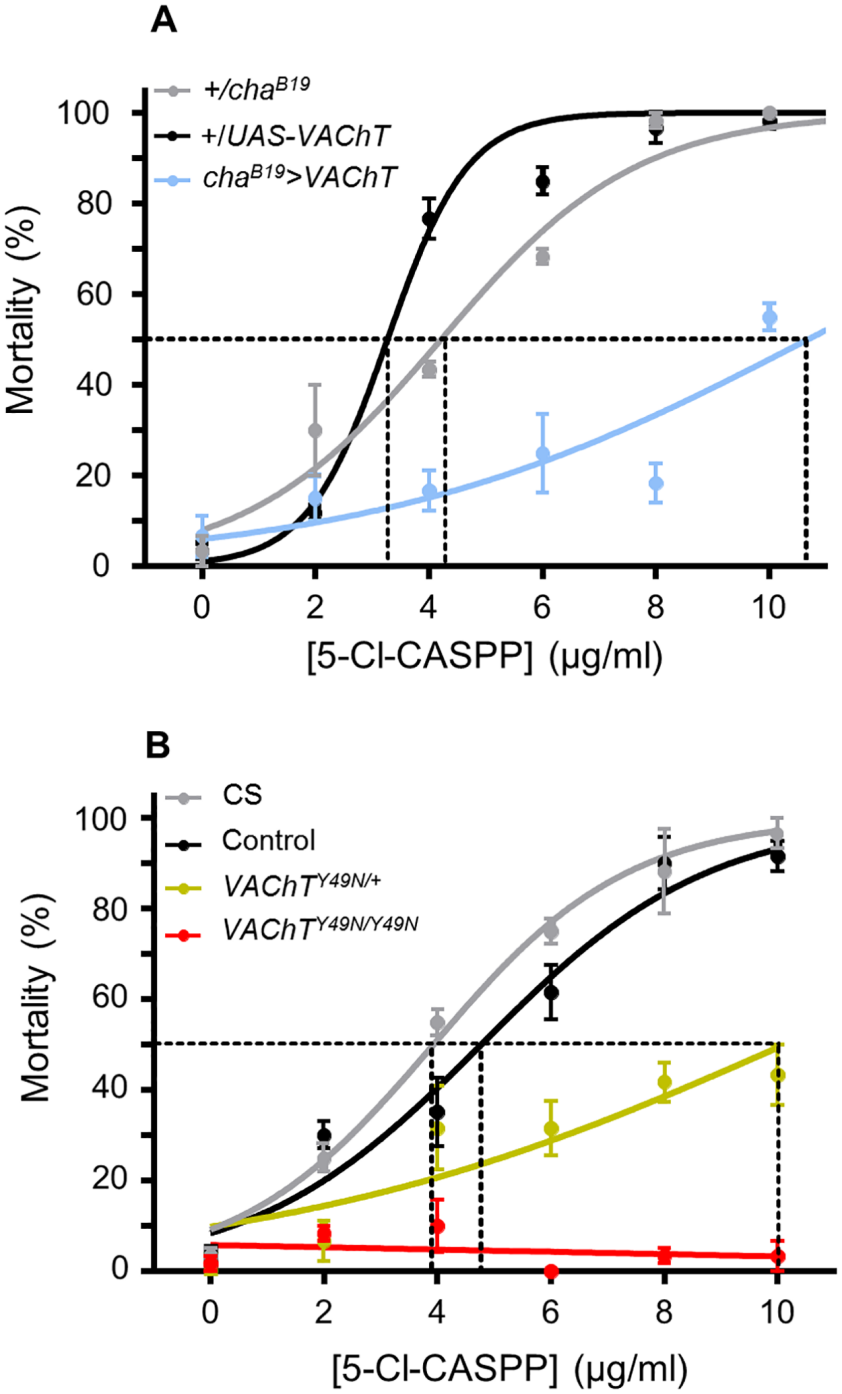
*VAChT* up-regulation or endogenous *VAChT*^*Y49N*^ provides resistance to 5-Cl-CASPP. (A) Mortality curves for +/UAS-*VAChT* (black), +/ *cha*^*B19*^ (grey) and *cha*^*B19*^>*VAChT* genotype larvae (blue) exposed to 5-Cl-CASPP (0–10 μg/ml). Transgenic expression of *VAChT* significantly (P = 1 x 10^−4^) reduced 5-Cl-CASPP toxicity compared with parental controls (LD_50_: 4.2 ± 0.2, 3.3 ± 0.1 vs. 10.7 ± 1.0 μg/ml, +/*cha*^*B19*^, +/*VAChT* and *cha*^*B19*^>*VAChT*, respectively). (B) Heterozygous *VAChT*^*Y49N*^ mutants (*VAChT*^*Y49N*/+^; yellow) show significantly reduced mortality to 5-Cl-CASPP compared with CS (grey) (LD_50_: 3.9 ± 0.2 vs. 10.1 ± 1.0 μg/ml, CS vs. *VAChT*^*Y49N*/+^ respectively, P = 1 x 10^−4^) and the CRISPR control genotype (black) (LD_50_: 4.8 ± 0.3, P = 1 x 10^−4^). Homozygous *VAChT*^*Y49N*^ mutants (red) display complete insensitivity to 5-Cl-CASPP at all concentrations tested. All data points are mean ± sem, n = 60 larvae tested at each concentration. Dotted lines represent LD_50_.

In agreement with previously published data [25], transgenic over-expression of wildtype *VAChT* significantly reduced 5-Cl-CASPP toxicity compared with parental controls (GAL4/+ and UAS/+). The observed LD_50_ values were: 4.2 ± 0.2, 3.3 ± 0.1 vs. 10.7 ± 1.0 μg/ml, +/*cha*^*B19*^, +/*VAChT* and *cha*^*B19*^>*VAChT* respectively, (P = 1 x 10^−4^, Fig 1A). Previously published work shows GAL4-mediated expression increases *VAChT* transcript abundance by ~2.8 fold [30]. Similarly, the presence of a single copy of *VAChT*^*Y49N*^, achieved by CRISPR-mediated gene replacement, provides resistance to 5-Cl-CASPP. Fig 1B shows heterozygous *VAChT*^*Y49N*/+^ larvae have significantly reduced mortality compared with CS (LD_50_: 3.9 ± 0.2 vs. 10.1 ± 1.0 μg/ml, CS vs. *VAChT*^*Y49N*/+^ respectively, P = 1 x 10^−4^). Remarkably, homozygous *VAChT*^*Y49N*^ mutants display a complete insensitivity to 5-Cl-CASPP at all concentrations tested (10ug/ml being the maximal practicable dose). QRT-PCR measurement of *VAChT*^*Y49N*^ transcript abundance shows that expression level does not differ to *VAChT* in control lines (1.3 ± 1.1 fold change, *VAChT*^*Y49N*/*Y49N*^ vs. combined control CS + CRISPR control, P = 0.37). It is noteworthy that the CRISPR control differs to CS in resistance to CASPP (LD_50_: 3.9 ± 0.2 vs. 4.8 ± 0.3, CS vs. CRISPR control, P = 0.02). However, *VAChT* transcript does not differ in level between CS and CRISPR control (P = 0.29), suggesting this additional resistance is likely due to differences in genetic background (which have not been controlled for). This difference does not, however, obscure the main observation that the presence of *VAChT*^*Y49N*^, at endogenous expression levels, provides complete resistance to 5-Cl-CASPP.

### Elevated *VAChT* increases frequency of spontaneous release events

**Fig 2:**
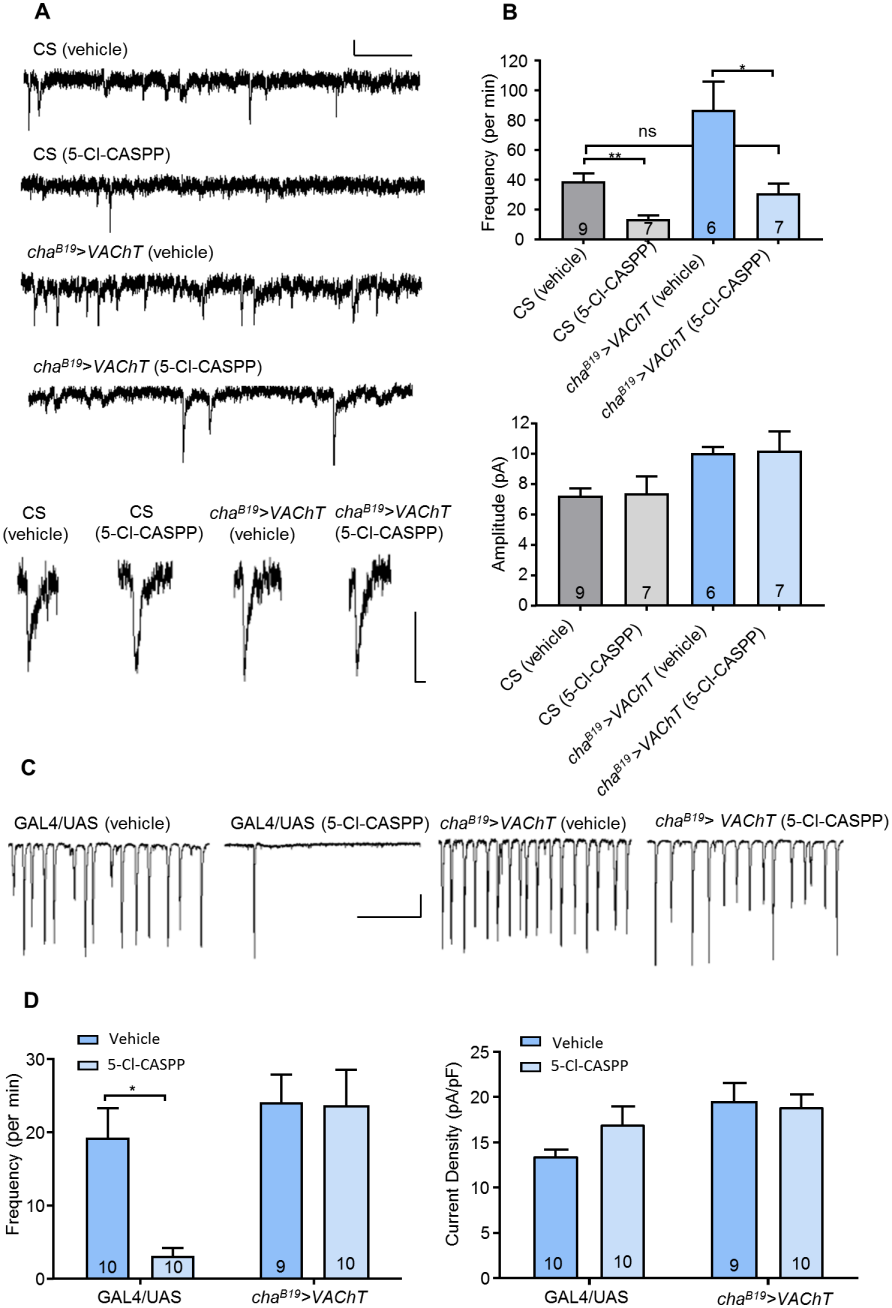
*VAChT* level modulates frequency of spontaneous release events. (A) Representative traces of minis recorded from L3 aCC/RP2 in either CS or *cha*^*B19*^>*VAChT* (fed acetone or 10 μg/ml 5-Cl-CASPP). Scale Bars (full trace: 10pA/20sec, single mini: 5pA/5ms) (B) 5-Cl-CASPP-dependent down-regulation of *VAChT* activity in CS (grey bars) results in a reduction in mini frequency (38.8 ± 5.6 vs. 13.2 ± 2.9 per minute, vehicle vs. 5-Cl-CASPP respectively, P = 2 x 10^−3^) with no effect to amplitude (P = 0.91). Exposure of a resistant background (blue bars, achieved by up-regulation of *VAChT*: *cha*^*B19*^>*VAChT*) to 5-Cl-CASPP also causes a significant reduction in mini frequency (86.8 ± 19.3 vs. 31.0 ± 6.3 per minute, vehicle vs. 5-Cl-CASPP respectively, P = 0.01). However, the reduction in absolute level of mini frequency is to a level not significantly different to CS (treated with vehicle). Exposure of the resistant background to 5-Cl-CASPP had no effect on mini amplitude (P = 0.94) (C) Representative traces of SRCs recorded from L3 aCC/RP2 in GAL4/+ and UAS/+ parental stocks and *cha*^*B19*^>*VAChT* (fed acetone or 10 μg/ml 5-Cl-CASPP). Scale Bar (400pA/20sec). (D) 5-Cl-CASPP dramatically reduces SRC frequency in parental controls (19.2 ± 4.1 vs. 3.1 ± 1.1 per min, vehicle vs. 5-Cl-CASPP respectively, P = 0.03) but with no effect to amplitude (P = 0.58). The effect of 5-Cl-CASPP on SRC frequency is rescued following up-regulation of *VAChT* (24.1 ± 3.8 vs. 23.5 ± 4.9, vehicle vs. 5-Cl-CASPP respectively, P = 1) with no obvious effect to amplitude (P = 1). All data points are mean ± sem, n stated in each bar.

To investigate the consequence for cholinergic synaptic function associated with observed 5-Cl-CASPP resistance, we undertook patch-clamp recordings from well-characterized aCC/RP2 motoneurons. These neurons receive identical cholinergic synaptic input [41]. We recorded spontaneous minis, achieved by blocking action potential-dependent activity with TTX. We have previously shown that exposure to 5-Cl-CASPP significantly reduces mini frequency, but does not affect mini amplitude [30]. In order to validate these data, and to provide a baseline for comparison with the experiments conducted as part of this study, we repeated this analysis. We observed the same results. 5-Cl-CASPP (10 μg/ml) mediated inhibition of *VACh*T results in a significant reduction in mini frequency (38.8 ± 5.6 vs. 13.2 ± 2.9 per min, vehicle vs. 5-Cl-CASPP respectively, P = 2 x 10^−3^, Fig 2A and 2B) but no effect to mini amplitude (P = 0.91, Fig 2A and 2B). By contrast, up-regulation of wildtype *VAChT* is sufficient to increase mini frequency, without change to amplitude [30]. Exposure of this genotype (*cha*^*B19*^>*VAChT*) to 5-Cl-CASPP also results in a significant reduction in mini frequency (86.8 ± 19.3 vs. 31.0 ± 6.3 per min, vehicle vs. 5-Cl-CASPP respectively, P = 0.01) and no change to amplitude (P = 0.94, Fig 2A and 2B). However, the reduction in absolute level of mini frequency, observed following exposure to CASPP, is to a degree that is not significantly different to untreated CS (treated with vehicle, P = 0.99).

Exposure to 5-Cl-CASPP also dramatically reduces the frequency of spontaneous rhythmic currents (SRCs), representative of action potential-dependant mass vesicle exocytosis (19.2 ± 4.1 vs. 3.1 ± 1.1 per min, vehicle vs. 5-Cl-CASPP respectively, P = 0.03) with no effect to SRC amplitude (P = 0.58, Fig 2C and 2D). This is suggestive of an inability of the cholinergic premotor interneurons to maintain wildtype evoked SVs release in the presence of 5-Cl-CASPP. The lack of effect to SRC amplitude, moreover, suggests that the premotor terminals release a relatively fixed number of SVs per release event: if the required number of ‘full’ SVs is not available, no evoked release seemingly occurs. The reduction in SRC frequency is rescued by the up-regulation of wildtype *VAChT* (24.1 ± 3.8 vs. 23.5 ± 4.9, vehicle vs. 5-Cl-CASPP respectively, P = 1), again with no obvious effect on SRC amplitude (P = 1). Taken together, these data suggest that upregulation of *VAChT* is sufficient to infer CASPP-resistance through maintenance of cholinergic SV release above a threshold level (approximately equal to that observed in CS/CRISPR control lines).

### Expression of *VAChT*^*Y49N*^ provides resistance to 5-Cl-CASPP through heightened SV release

**Fig 3:**
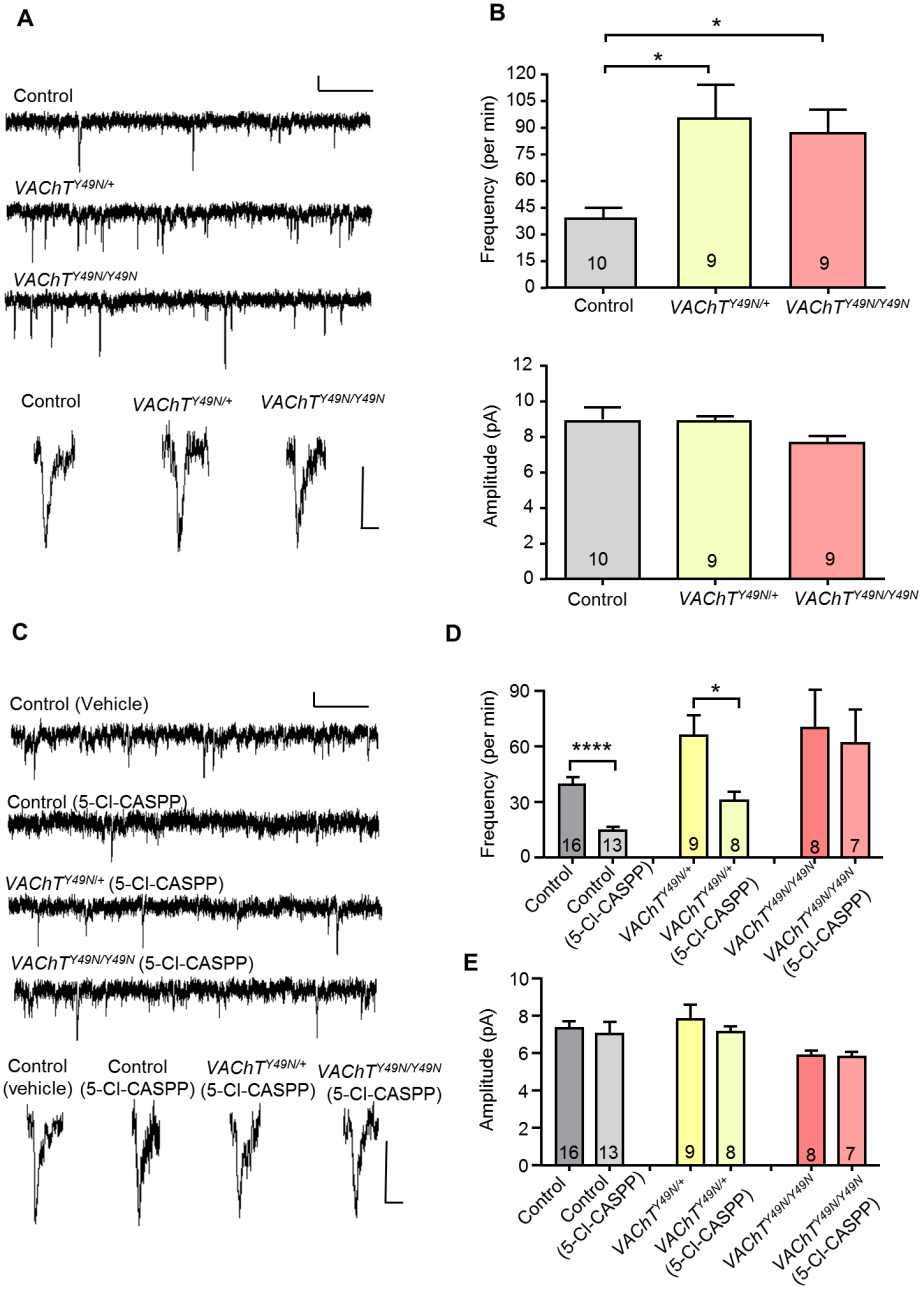
*VAChT*^*Y49N*^ increases mini frequency. (A) Representative traces of cholinergic minis from L3 aCC/RP2 in control (CS + CRISPR control combined, CS presented in trace), *VAChT*^*Y49N*/+^ and *VAChT*^*Y49N*/*Y49N*^ genotypes. Scale Bars (full trace: 10pA/20sec, single mini: 5pA/5ms). (B) *VAChT*^*Y49N*^ mutants show a significant increase in mini frequency, compared to control, in both heterozygotes (41.2 ± 6.8 vs. 95.2 ± 19.1 per min, P = 0.02) and homozygotes (87.2 ± 13.3 per min, P = 0.04). However, no effect was observed to mini amplitude (P = 0.32). (C) Representative traces of minis recorded from L3 aCC/RP2 in control (CS and CRISPR control lines combined), *VAChT*^*Y49N*/+^ and *VAChT*^*Y49N*/*Y49N*^ fed either acetone or 10 μg/ml 5-Cl-CASPP. Scale Bars (full trace: 10pA/20sec, single mini: 5pA/5ms). (D) Mini frequency in controls is significantly reduced following ingestion of 10 μg/ml 5-Cl-CASPP as opposed to those fed vehicle only (38.8 ± 4.2 vs. 14.3 ± 1.8 per min, vehicle vs. 5-Cl-CASPP respectively, P = 1 x 10^−4^). Mini frequency is also significantly reduced in heterozygous *VAChT*^*Y49N*/+^ (65.2 ± 10.8 vs. 30.3 ± 4.8 per min, vehicle vs. 5-Cl-CASPP respectively, P = 0.01) but to a level that is not significantly different from control values fed vehicle (38.8 ± 4.2 vs. 30.3 ± 4.8 per min, P = 1). Homozygosity of *VAChT*^*Y49N*/*Y49N*^ is sufficient to completely prevent 5-Cl-CASPP reducing mini frequency (69.3 ± 20.5 vs. 61.0 ± 18.1 per min, vehicle vs. 5-Cl-CASPP respectively, P = 0.78). (E) No significant change in mini amplitude was recorded for control (P = 0.67) *VAChT*^*Y49N/+*^ (P = 0.49) or *VAChT*^*Y49N/Y49N*^ (P = 0.82). All data points are mean ± sem, n stated in each bar.

Genomic replacement of *VAChT*^*49Y*^ by *VAChT*^*49N*^ increases mini frequency (41.2 ± 6.8 vs. 95.2 ± 19.1 vs. 87.2 ± 13.3 per min, control (CS + CRISPR control combined), *VAChT*^*Y49N/+*^, *VAChT*^*Y49N/Y49N*^, respectively, P = 0.02 and 0.04, Fig 3A and 3B). By contrast, no effect on mini amplitude was observed, either for heterozygous or homozygous *VAChT*^*Y49N*^ (Fig 3B). The observed resistance to 5-Cl-CASPP induced by expression of *VAChT*^*Y49N*^ may thus be due to the maintenance of cholinergic mini release frequency above a critical threshold value (as described above). To test this we measured mini frequency and amplitude in the presence of 5-Cl-CASPP. As expected, mini frequency was significantly reduced in controls following ingestion of 5-Cl-CASPP (10 μg/ml, 38.8 ± 4.2 vs. 14.3 ± 1.8 per min, P = 1 x 10^−4^, Fig 3C and 3D). Mini frequency was also significantly reduced in the heterozygous *VAChT*^*Y49N*/+^ following exposure to 5-Cl-CASPP (65.2 ± 10.8 vs. 30.3 ± 4.8 per min, vehicle vs. 5-Cl-CASPP respectively, P = 0.01), but to a level that was not significantly different to controls fed vehicle (38.8 ± 4.2 vs. 30.3 ± 4.8 per min, P = 1). Homozygous expression of *VAChT*^*Y49N*/*Y49N*^ is, however, sufficient to completely prevent 5-Cl-CASPP from reducing mini frequency (69.3 ± 20.5 vs. 61.0 ± 18.1 per min, vehicle vs. 5-Cl-CASPP respectively, P = 0.78). No significant change in mini amplitude was observed for any treatment (Fig 3E). These data are consistent with *VAChT*^*Y49N*^ providing resistance to 5-Cl-CASPP through maintenance of a threshold frequency of cholinergic mini release. Moreover, resistance would appear proportional to mini release frequency.

### Expression of *Sytx*^*3-69*^ or *cpx*^*SH1*^ fails to provide resistance to 5-Cl-CASPP

To validate the hypothesis that spontaneous release frequency dictates resistance to 5Cl-CASPP, we identified two additional genetic approaches reported to elevate glutamatergic minis at the *Drosophila* NMJ. Larval mortality assays and mini recordings from aCC/RP2 were conducted following expression of *sytx*^*3-69*^ or in a *complexin* null mutant, *cpx*^*SH1*^ [37, 38].

**Fig 4:**
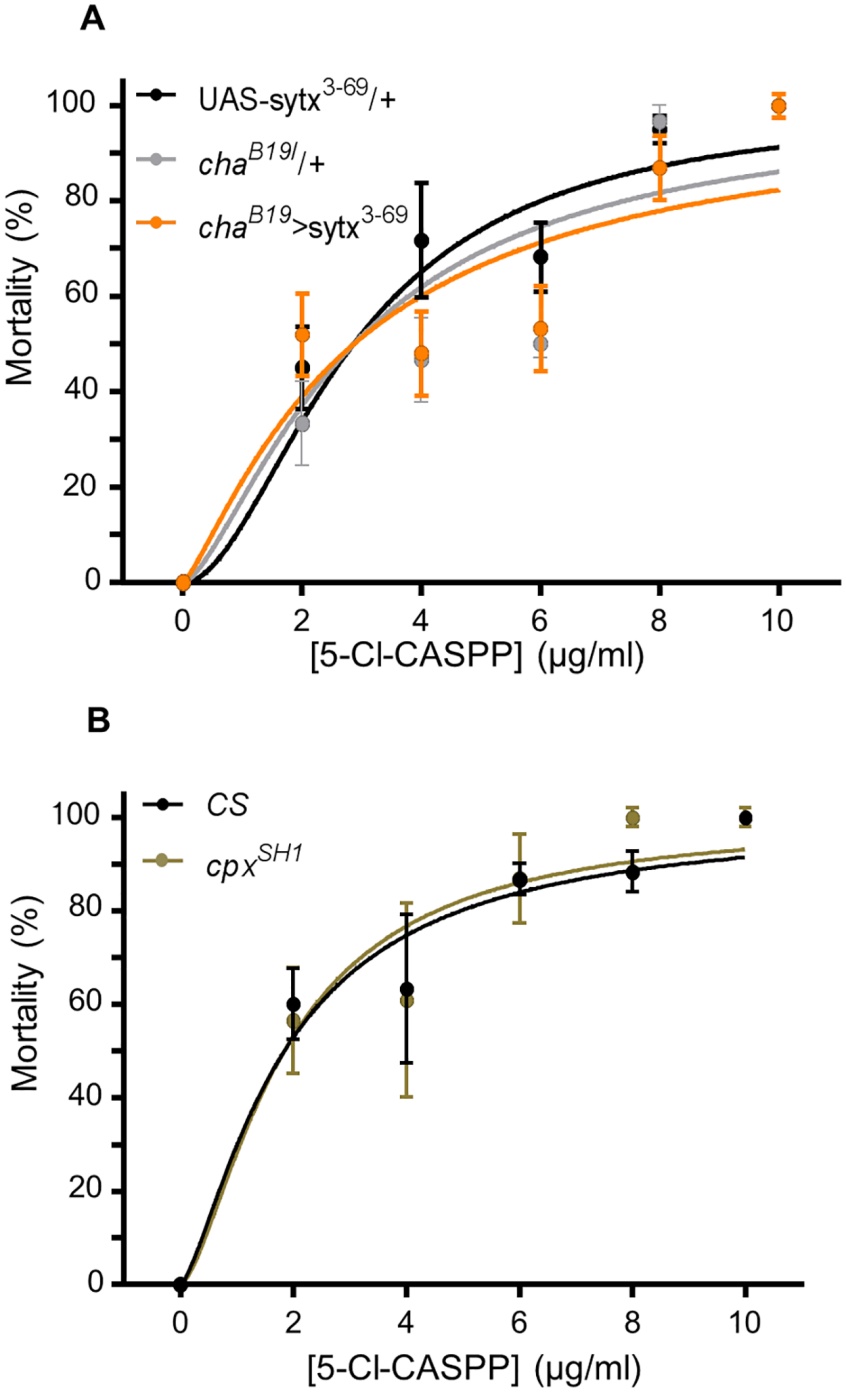
Alternative routes that maintain mini release fail to provide resistance to 5-Cl-CASPP. (A) Expression of *sytx*^*3-69*^ in cholinergic neurons is sufficient to increase mini frequency (see text) but does not afford resistance to 5-Cl-CASPP. The observed LD_50_ values were: 3.8 ± 0.5, 2.6 ± 0.3 vs. 2.4 ± 0.4 μg/ml, +/*cha*^*B19*^ (n = 60 larvae tested per concentration), +/*sytx*^*3-69*^ (n = 60 per concentration) vs. *cha*^*B19*^> *sytx*^*3-69*^ (n = 80 per concentration) respectively, P = 0.07). (B) The *cpx*^*SH1*^ mutant similarly has no resistance to 5-Cl-CASPP. Observed LD_50_ values in this case were: 1.7 ± 0.2 vs. 2.0 ± 0.3, CS vs. *cpx*^*SH1*^ respectively, n = 60 per concentration, P = 0.73). All data points shown are mean ± sem.

Expression of *sytx*^*3-69*^ in cholinergic premotor interneurons (*cha*^*B19*^>*sytx*^*3-69*^) dramatically increased mini frequency in aCC/RP2 (43.0 ± 15.9 vs. 489.8 ± 115.1 per min, GAL4/+ vs. *cha*^*B19*^>*sytx*^*3-69*^ respectively, P = 5 x 10^−3^). In agreement with previous data, exposure to 5-Cl-CASPP significantly reduced mini frequency in this genotype (489.8 ± 115.1 vs. 201.4 ± 45.1 per min, vehicle vs. 5-Cl-CASPP respectively, P = 0.02), but to a final level ~4.5-fold greater than controls. However, expression of *sytx*^*3-69*^ afforded no resistance to 5-Cl-CASPP. Observed LD_50_ values to CASPP were: 3.8 ± 0.5, 2.6 ± 0.3 vs. 2.4 ± 0.4 μg/ml, +/*cha*^*B19*^, +/*sytx*^*3-69*^ vs. *cha*^*B19*^> *sytx*^*3-69*^ respectively, P = 0.07, Fig 4A).

The *complexin* null mutant (*cpx*^*SH1*^) similarly increased mini release compared to that observed in CS wildtype (43.3 ± 7.2 vs. 100.4 ± 13.5 per min, CS vs. *cpx*^*SH1*^ respectively, P = 4 x 10^−3^). Interestingly, 5-Cl-CASPP did not significantly reduce mini frequency in this mutant (100.4 ± 13.5 vs. 81.9 ± 17.7 per min, vehicle vs. 5-Cl-CASPP respectively, P = 0.58). Moreover, despite the apparent maintenance of heightened mini release, this did not precipitate any obvious effect to larval survival when challenged with 5-Cl-CASPP. Observed LD_50_ values were: 1.7 ± 0.2 vs. 2.0 ± 0.3 μg/ml, CS vs. *cpx*^*SH1*^ respectively, P = 0.73, Fig 4B). These data suggest that although a reduction in spontaneous mini frequency is correlated with CASPP-induced toxicity, maintenance of this mode of synaptic release does not infer resistance. We therefore looked towards how *VAChT*^*Y49N*^ resistance to CASPP affects action potential-evoked activity (i.e. SRCs).

### *VAChT*^*Y49N*^ disrupts rhythmicity of cholinergic circuits

To assess how *VAChT*^*Y49N*^ influenced motoneuron activity, we examined endogenous bursting activity (as measured by loose patch) of aCC/RP2 motoneurons. The advantage of loose patch is that the interior of the cell is not affected (the membrane remains intact). We measured both frequency of bursts and the number of individual action potentials (APs) per burst. Previous work has shown that a single SRC gives rise to a sustained depolarization (burst) that elicits multiple APs in motoneurons (22). Thus, burst frequency represents the evoked release of ACh from premotor interneurons, whilst APs fired is a postsynaptic property of the motoneurons. CS larvae exhibited 27.9 ± 7.5 bursts per minute, with an average of 15.0 ± 1.8 APs per burst. By contrast, *VAChT*^*Y49N/Y49N*^ showed significantly reduced burst frequency (9.0 ± 4.2 per min, P = 0.04). This is predictive of a decreased ability of the premotor interneurons to maintain a normal synaptic output. However, APs per burst remained constant (12.8 ± 3.0 spikes per burst, P = 0.54) indicative that the postsynaptic motoneuron is not affected. Thus, despite an increase in spontaneous mini release (cf. Fig 3B), these data show that the *VAChT*^*Y49N*^ point mutation does not support ‘normal’ presynaptic cholinergic input to motoneurons which, in turn, disrupts motoneuron burst frequency. However, the number of APs fired by motoneurons per burst is unaffected.

**Fig 5:**
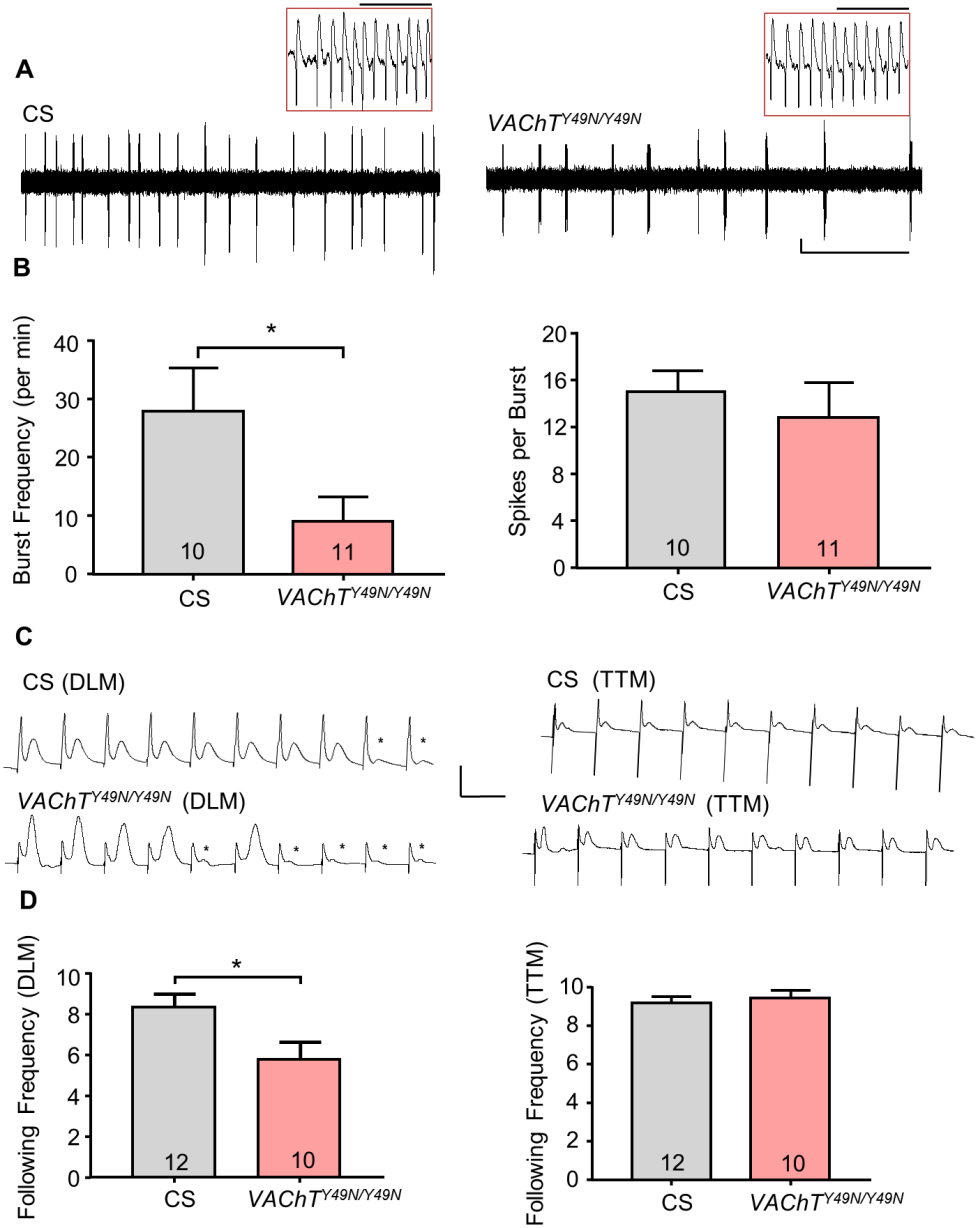
*VAChT*^*Y49N*^ disrupts cholinergic synaptic inputs to motor neurons. (A) Representative traces of extracellular bursting activity recorded from L3 aCC/RP2 in CS or *VAChT*^*Y49N/Y49N*^. Scale Bar (1mV/15sec). A single burst is also shown at an expanded time scale for each recording, inset: 50ms). (B) *VAChT*^*Y49N/Y49N*^ show a significant reduction in endogenous bursting (27.9 ± 7.5 vs. 9.0 ± 4.2 per min, CS vs. *VAChT*^*Y49N/Y49N*^ respectively, P = 0.04). However, number of spikes per burst remains consistent (15.0 ± 1.8 vs. 12.8 ± 3.0 spikes per burst, P = 0.54). (C) Representative traces of adult GFS recordings from DLM/TTM in either CS or *VAChT*^*Y49N/Y49N*^. Scale Bar (30mV/10ms). (D) During high frequency stimulation (100Hz), the DLM in *VAChT*^*Y49N/Y49N*^ are less able to follow a train of 10 stimuli (8.3 ± 0.7 vs. 5.8 ± 0.9, CS vs. *VAChT*^*Y49N/Y49N*^ respectively, P = 0.03). Dots show synaptic failures. The TTM remains unaffected (9.2 ± 0.3 vs. 9.4 ± 0.4 CS vs. *VAChT*^*Y49N/Y49N*^ respectively, P = 0.60). All data points are mean ± sem, n stated in each bar.

We next asked whether changes to evoked cholinergic synaptic release are observable in other cholinergic neural circuits. We used the adult giant fibre system (GFS). The physiological output from stimulation of the adult GF is muscular contraction of both the tergotrochanteral muscle (TTM) and the dorsal longitudinal flight muscle (DLM) [43]. Although both DLMs and TTMs are directly innervated by glutamatergic synapses, TTM motoneurons are excited via both cholinergic and electrical synapses, whereas DLM motoneurons are excited solely be cholinergic synapses. We find that, during high frequency stimulation (100Hz), the DLM of *VAChT*^*Y49N*^ is less able to follow a train of 10 stimuli (8.3 ± 0.7 vs. 5.8 ± 0.9, CS vs. *VAChT*^*Y49N*^ respectively, P = 0.03, Fig 5C and 5D). The TTM, by contrast, which has additional electrical synapses, remains unaffected (9.2 ± 0.3 vs. 9.4 ± 0.4 CS vs. *VAChT*^*Y49N*^ respectively, P = 0.60). These data further support the hypothesis that the *VAChT*^*Y49N*^ mutation disrupts the ability of cholinergic neurons to maintain sustained evoked release.

### The *VAChT*^*Y49N*^ resistance mutation disrupts evoked vesicle release

**Fig 6:**
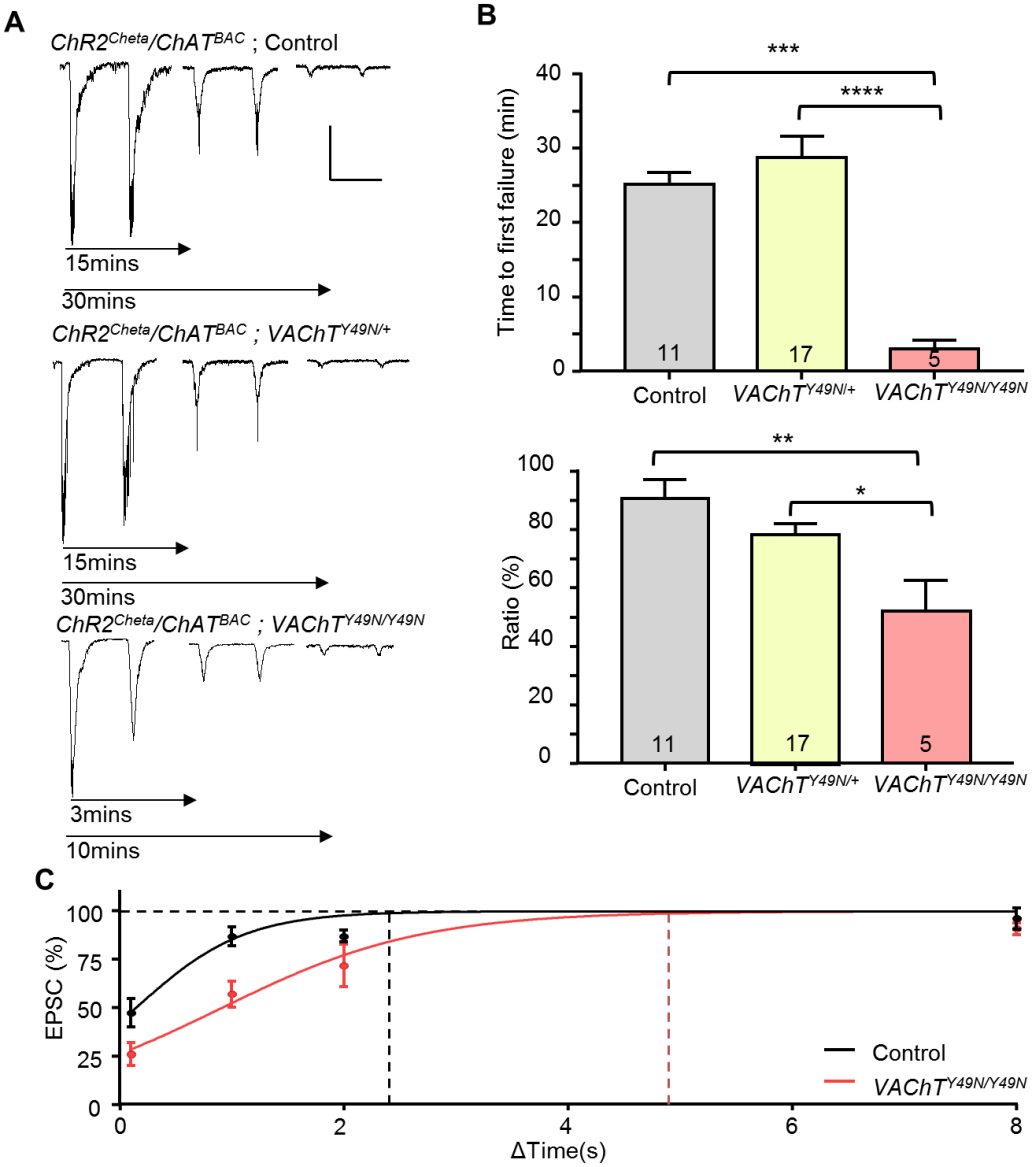
*VAChT*^*Y49N*^ disrupts evoked cholinergic release. (A) Representative traces of ChR-activated EPSCs recorded from L3 aCC/RP2 in control, *VAChT*^*Y49N/+*^ and *VAChT*^*Y49N/Y49N*^. Scale Bar (100pA/1sec) (B) At a stimulation frequency of 1Hz, homozygous *VAChT*^*Y49N/Y49N*^ mutants show a significantly reduced time until first synaptic failure compared to controls (25.1 ± 1.6 vs. 0.9 ± 0.4 minutes, control vs. *VAChT*^*Y49N/Y49N*^ respectively, P = 2 x 10^−3^). This phenotype is absent in the heterozygote (25.1 ± 1.6 vs. 28.7 ± 2.9 minutes, control vs. *VAChT*^*Y49N/+*^, P = 1). The ratio change in current density between the 1st and 2nd EPSC is also significantly reduced (90.4 ± 6.5 vs. 48.1 ± 17.8%, control vs. *VAChT*^*Y49N/Y49N*^ respectively, P = 0.02). (C) Percentage (2/1) EPSC amplitude at time intervals 0.1, 1, 2 and 8 seconds following initial stimulation between control (n = 7, 16, 5, 5 respectively) and *VAChT*^*Y49N/Y49N*^ (n = 10, 10, 5, 5 respectively). The minimum time interval required between successive stimulations to generate a second full size EPSC at 100% in *VAChT*^*Y49N/Y49N*^ is approximately doubled compared with control lines (2.4 vs. 4.8s, control vs. *VAChT*^*Y49N/Y49N*^ respectively). All data points are mean ± sem, n stated in each bar.

A reduction in burst frequency of aCC/RP2 in homozygous *VAChT*^*Y49N*^ is indicative of an inability to maintain a normal presynaptic cholinergic release. In order to address this question, we tested the ability of motoneurons to follow prolonged stimulation of premotor cholinergic interneurons. We used the blue light-sensitive *ChR* variant, *UAS-ChR*^*ChETA*^, expressed in all cholinergic neurons (*ChAT*^*BAC*^ GAL4), to allow us to stimulate presynaptic activity. Long term stimulation at 1Hz in control lines (CS + CRISPR control) or *VAChT*^*Y49N/+*^ resulted in a gradual decline in evoked EPSC amplitude (these EPSPs are equivalent to SRC’s but are evoked by optogenetic stimulation). Full failure of transmission occurred at 25.1 ± 1.6 vs. 28.7 ± 2.9 min (control vs. *VAChT*^*Y49N/+*^, P = 1, Fig 6A and 6B). By contrast, EPSC amplitude falls off dramatically after the first stimulation in homozygous *VAChT*^*Y49N*^ (failure of transmission occurring at 2.9 ± 1.3 min, P = 3 x 10^−4^). A reduction in successive EPSC amplitude is clearly observable between the 1^st^ and 2^nd^ induced EPSCs, which is significantly greater in *VAChT*^*Y49N/Y49N*^ (90.4 ± 6.5 vs. 52.0 ± 10.6%, control vs. *VAChT*^*Y49N/Y49N*^ respectively, P = 2 x 10^−3^). Thus, although the amplitude of the first EPSC is not different, the ability to support a full-size second EPSC is significantly compromised in *VAChT*^*Y49N*^. Fig 6C displays ratio (%) of EPSC amplitude difference (2/1) at time intervals 0.1, 1, 2 and 8 seconds following initial stimulation. Regression analysis suggests the minimum time interval required between successive stimulations to generate a second full size EPSC in *VAChT*^*Y49N/Y49N*^ is approximately doubled compared with control lines (2.4 vs. 4.8 s, control vs. *VAChT*^*Y49N/Y49N*^ respectively). This is entirely consistent with the reduced frequency of burst activity recorded in these same motoneurons by loose-patch (c.f. Fig 5A and 5B).

### Insecticide resistance and other measures of fitness

**Fig 7:**
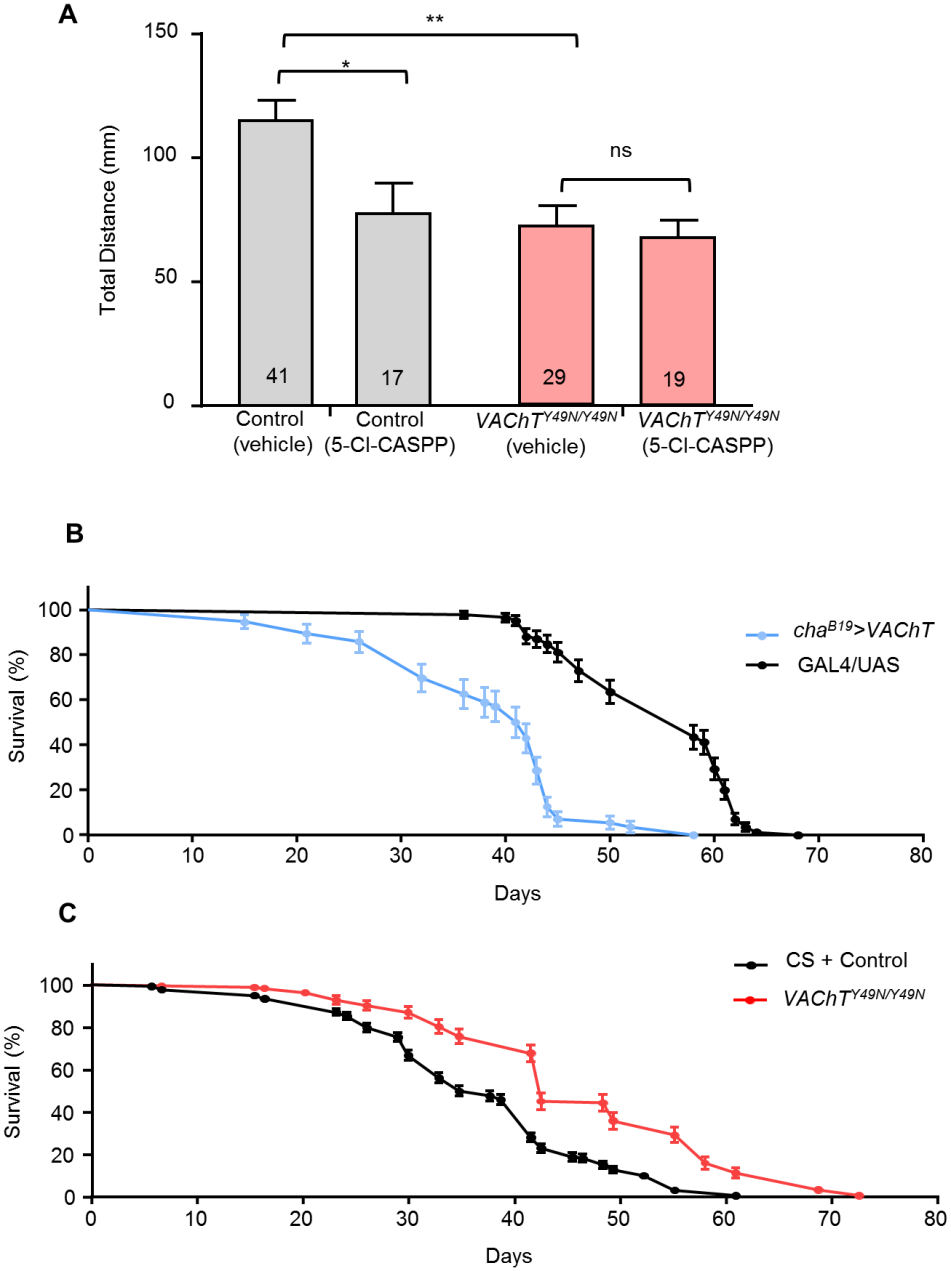
*VAChT*^*Y49N*^ disrupts larval locomotion. (A) 5-Cl-CASPP treatment (5 μg/ml) significantly reduces total distance moved (116.5 ± 8.5 vs. 78.6 ± 12.1 mm, control: vehicle vs. 5-Cl-CASPP respectively, P = 0.04) in control L3. Reduced distance (116.5 ± 8.5 vs. 72.7 ± 8.3 mm, vehicle: control vs. *VAChT*^*Y49N/Y49N*^ respectively, P = 2 x 10^−3^) was also observed in *VAChT*^*Y49N*^ (0.65 ± 0.05 vs. 0.41 ± 0.05 mm/s, P = 2 x 10^−3^) in the absence of treatment. However, exposure of *VAChT*^*Y49NY49N*^ to 5-Cl-CASPP does not further affect locomotion (72.7 ± 8.3 vs. 68.3 ± 7.1 mm, *VAChT*^*Y49N/Y49N*^: vehicle vs. 5-Cl-CASPP respectively, P = 1). (B) Transgenic overexpression of *VAChT* (*cha*^*B19*^*>VAChT*, light blue) elicits a reduction in adult longevity (median survival: 41.5 vs. 58 days, *cha*^*B19*^>*VAChT* (n = 56) vs. GAL4/UAS (n = 85) respectively, P = 1 x 10^−4^). (C) Homozygous *VAChT*^*Y49N*^ (red) adults show increased longevity (median survival: 36.0 vs. 44.0 days, merged controls (CS + CRISPR control) (n = 400) vs. *VAChT*^*Y49N/Y49N*^ (n = 150) respectively, P = 1 x 10^−4^). All data points are mean ± sem, n stated in each bar or given as individual data points.

We have shown that *VAChT*^*Y49N*^ perturbs endogenous cholinergic activity. Given the reliance on this neurotransmitter for locomotion, a significant change in behaviour might be expected. We therefore examined larval crawling behaviour. A low 5-Cl-CASPP treatment of 5μg/ml was used for crawling assays in order to prevent complete paralysis (observed in control lines at higher CASPP exposure). We found that 5-Cl-CASPP treatment of control (CS + CRISPR control) larvae significantly reduced total distance travelled (116.5 ± 8.5 vs. 78.6 ± 12.1 mm, control: vehicle vs. 5-Cl-CASPP respectively, P = 0.04, Fig 7A). The presence of *VAChT*^*Y49N*^ similarly reduces larval total distance (116.5 ± 8.5 vs. 72.7 ± 8.3 mm, vehicle: control vs. *VAChT*^*Y49N/Y49N*^ respectively, P = 2 x 10^−3^, Fig 7A). However, *VAChT*^*Y49N*^ larvae were not further affected when fed 5-Cl-CASPP (72.7 ± 8.3 vs. 68.3 ± 7.1 mm, *VAChT*^*Y49N/Y49N*^, vehicle vs. 5-Cl-CASPP respectively, P = 1, Fig 7A).

Transgenic overexpression of wildtype *VAChT* (*cha*^*B19*^*>VAChT*) elicits a dramatic reduction in adult longevity (median survival: 58.0 vs. 41.5 days, GAL4/UAS vs. *cha*^*B19*^>*VAChT* respectively, P = 1 x 10^−3^, Fig 7B). The associated hazard ratio of 4.06 indicates that overexpression of *VAChT* results in a 4-fold reduction in survival probability, at any point throughout the longevity assay. Conversely, fly lines homozygous for *VAChT*^*Y49N*^ exhibit increased longevity (median survival: 36.0 vs. 44.0 days, control vs. *VAChT*^*Y49N/Y49N*^ respectively, P = 1 x 10^−3^, Fig 7C). In this experiment control lines suffered a hazard ratio of 2.4 relative to *VAChT*^*Y49N*^. CS and CRISPR control fly lines did not differ in longevity (median survival: 43.0 vs. 34.0 days, CS vs. CRISPR control respectively, P = 0.07, Fig 7C) and were therefore combined for comparison to *VAChT*^*Y49N/Y49N*^.

Finally, we measured fecundity in the form of oviposition rate of newly-eclosed mated females. Average oviposition per female remained remarkably similar between all genotypes in either *VAChT* upregulation (40.7 ± 5.6 vs. 31.2 ± 5.4, GAL4/UAS vs. *cha*^*B19*^*>VAChT* respectively, P = 0.26) or *VAChT*^*Y49N/Y49N*^ (34.2 ± 4.1 vs 33.2 ± 6.6, control vs. *VAChT*^*Y49N/Y49N*^ respectively, P = 0.90).

## Discussion

We report neurophysiological consequences resulting from two potential mechanisms of CASPP-resistance. Resistance obtained through *VAChT* up-regulation correlates to an increase in the availability and/or probability of SV release and not through alteration of the amount of neurotransmitter loaded per vesicle (i.e. quantal size). The lower bounds of sustainable activity appear to be dictated by a minimum threshold for release frequency, below which normal cholinergic release is unsustainable and the likelihood of mortality increases. However, resistance associated through the second route, endogenous expression of the point mutation *VAChT*^*Y49N*^, although showing a similar spontaneous release profile, further uncovers significant deficiencies in evoked neurotransmitter release. We attribute this to an inability of the point mutation to maintain sustained evoked SV release, perhaps indicative of a fitness trade-off between protein functionality and insecticide resistance.

Insecticide resistance alleles commonly produce negative effects on multiple life-traits (including larval development, sex ratio, oviposition/fecundity, mass, embryonic viability and adult longevity) [44–46] and in some cases these are proportional to resistance level [46]. However, exceptions have been reported suggesting interactions between resistance modality and target protein functionality dictates complex life-traits [47]. We show in this study that although resistance may be attained through *VAChT* up-regulation, adult longevity is reduced. Reduced lifespan is also associated with transgenic up-regulation of *VGLUT*, where adult male longevity is reduced by >50% [18]. *VGLUT* mediated excitotoxicity and neurodegeneration may result from increased quantal size (i.e. excess glutamate release) [18, 48]. Although, similar post-synaptic degeneration may occur in central cholinergic neurons following up-regulation of *VAChT*, the degree of excitotoxicity is predicted to be lower due to the inability of this transporter to affect quantal size (only affecting release frequency) [30]. Cash et al show that, in aCC motoneurons, either *VAChT* up-regulation or pharmacological inhibition of *VAChT* through 5-Cl-CASPP treatment is not sufficient to alter the postsynaptic response to perfused ACh [30]. However, up-regulation of the *nAChR*^*D7*^ subunit does show an increase in postsynaptic response to perfused ACh [30], suggesting that postsynaptic receptor kinetics can be modulated at central cholinergic synapses, however, not through genetic or pharmacological manipulation of pre-synaptic VAChT activity. Given the similarities in spontaneous release kinetics between *VAChT* up-regulation and the *VAChT*^*Y49N*^ mutation, together with the observation that the Y49N mutation reduces endogenous and evoked activity, we rationalize it unlikely that the Y49N point mutation or VAChT up-regulation would cause a consequential alteration in postsynaptic kinetics or precipitate acute cholinergic neurotoxicity. Following a similar logic, the reduced evoked release of SVs (i.e. SRCs) in *VAChT*^*Y49N*^ may underlie the increased longevity observed in this genotype; reduced transmission being protective. The inability to influence quantal size, at central cholinergic synapses, through up-regulation of *VAChT* is indicative that vesicle-loading obeys the proposed “set-point” SV loading model [30, 49]. This model dictates that the level to which SVs are filled is pre-set and, thus, cannot be influenced through an increased loading rate (which might be expected following increased transporter expression). Several other studies are in agreement with this conclusion [30, 50]. However, studies at mouse, rat and nematode NMJs link modulation of *VAChT* activity with corresponding changes in quantal size; conforming to the steady-state SV filling model [14, 51–53]. Furthermore, increased *VGLUT* at glutamatergic synapses at the *Drosophila* NMJ is sufficient to increase quantal size through the manipulation of vesicle size [48], an effect also not observed following up-regulation of *VAChT* expression [30]. Collectively, these studies are indicative of a significant difference between either SV loading at central vs. peripheral synapses and/or differences between cholinergic and glutamatergic synapses.

Our findings in this study are indicative of a direct relationship between SV release probability and pharmacological and/or genetic regulation of *VAChT*. Our data suggest, moreover, that block of *VAChT* through CASPP, results in a reduction in SV release frequency. This finding agrees with previously published data [30] and correlates with CASPP lethality [25]. Up-regulation of *VAChT* or expression of *VAChT*^*Y49N*^ mediates resistance to CASPP, an effect that is associated with the maintenance of spontaneous SV release above a critical threshold. The resistance effect of up-regulation of wildtype *VAChT* could be simply explained by an increase in the number of vesicles that escape block of *VAChT* by CASPP and are thus able to reach set-point. The resistance effect of replacing the wildtype *VAChT* with the Y49N mutant form however, might be explained by a combination of different factors: Expression of *VAChT*^*Y49N*^ may confer resistance to CASPP because binding of the insecticide to the transporter is prevented, because binding of CASPP to *VAChT*^*Y49N*^ no longer prevents ACh transport and therefore does not critically reduce the number of vesicles reaching set point, and/or because binding and inhibition of *VAChT*^*Y49N*^ function by CASPP is compensated by an increased release probability conferred by the Y49N mutation. Further experiments would be required to confirm or rule out the individual contributions of these factors.

The expression of *VAChT*^*Y49N*^ additionally disrupts the ability of the cholinergic system to maintain sustained evoked release. This is evidenced by a reduction in frequency of endogenous bursts of AP firing in motoneurons and by a failure of the DLM to follow high frequency stimulation. The mechanism underlying this may be heightened spontaneous mini release, which in turn reduces the pool of SVs available for evoked release. This conclusion is in agreement with work at the glutamatergic *Drosophila* NMJ which reports *complexin* null mutants display a reduction in EPSPs (equivalent of SRCs) together with increased mini frequency [38]. However, the syntaxin mutant (*syx*^*3-69*^) and a SNAP-25 mutant (*SNAP25*^*ts*^) maintain higher mini frequency together with EPSPs that are increased in amplitude [37, 54].

CRISPR-dependent expression of *VAChT*^*Y49N*^ is sufficient to increase frequency of minis and, also, to prevent a normal pattern of evoked release of ACh from premotor cholinergic interneurons. Only the former results following the up-regulation of wildtype *VAChT* level. In addition to suggesting that the contribution of this transporter to spontaneous and evoked release may be separable, it identifies a region of the transporter that is seemingly required for sustained evoked release. The *VAChT*^*Y49N*^ mutation disrupts a region resembling the well-characterized trafficking motif YXXØ (with Ø being any large hydrophobic amino acid): specifically causing a YMVI to NMVI mutation. This motif, amongst others (e.g. YNYY in mammals) is known to be involved in protein sorting, the Y residue being highlighted as the most critical determinant [55]. The positioning of this signal on a cytosolic region of *VAChT* indicates that this region could potentially be involved in trafficking. Disruption of the YNYY motif in mammalian *VAChT* is sufficient to cause mis-localisation and retention of the transporter to the plasma membrane [56, 57]. Whilst mis-localisation or membrane retention of *VAChT* may be sufficient to perturb sustained evoked release, it does not offer a plausible explanation why spontaneous vesicle release is increased in these mutants and following up-regulation of wildtype *VAChT*. It is notable in this regard that a glycine-to-arginine substitution at position 347 (G347R) disrupts a reported *VAChT*-synaptobrevin interaction in *C*. *elegans* that impacts SV release probability [58]. Glycine is conserved in *Drosophila VAChT* at position 342 suggesting a potential similar involvement in the SNARE assemblage.

Up-regulation of genes relating to insecticide detoxification (e.g. glutathione S-transferase and cytochromes P450s) is a common form of insecticide resistance [59–62]. Natural expressional modification of target proteins (expression level or mutation) are rarer but have been found to confer resistance in other cholinergic targets. For example, upregulation of acetylcholinesterase (*ace-1)* is reported in organophosphate and pyrethroid resistant field populations in the spider mite *(Tetranychus evansi)* [63] in addition to gene duplication of *ace*-1 in mosquito (*Culex pipiens*) [64]. This study shows that expressional modification of a target protein (*VAChT*) or the acquisition of a point-mutation (Y49N) is sufficient to confer resistance to CASPP. This mutation affects a characterized motif in *VAChT* (YMVI) that is seemingly critical for high frequency SV evoked release. Further characterisation of this motif may provide a better understanding of how this transporter contributes to evoked synaptic release, but may also identify a novel target for insecticidal attack.
